# Factors Involved in the Functional Motor Recovery of Rats with Cortical Ablation after GH and Rehabilitation Treatment: Cortical Cell Proliferation and Nestin and Actin Expression in the Striatum and Thalamus

**DOI:** 10.3390/ijms20225770

**Published:** 2019-11-16

**Authors:** Margarita Heredia, Natalia Rodríguez, Virginia Sánchez Robledo, José María Criado, Antonio de la Fuente, Jesús Devesa, Pablo Devesa, Adelaida Sánchez Riolobos

**Affiliations:** 1 Department of Physiology and Pharmacology, Institute of Neurosciences of Castilla and León (INCyL), University of Salamanca, Avenida Alfonso X El Sabio s/n, 37007 Salamanca, Spain; natalia.rguez.gil@gmail.com (N.R.); robledo@usal.es (V.S.R.); jmcriado@usal.es (J.M.C.); jfuente@usal.es (A.d.l.F.); asriolob@usal.es (A.S.R.); 2 Scientific Direction, Medical Center Foltra, Travesía de Montouto 24, 15894 Teo, Spain; 3 Research and Development, Medical Center Foltra, Travesía de Montouto 24, 15894 Teo, Spain; pdevesap@foltra.org

**Keywords:** growth hormone, traumatic brain injury, neural plasticity, neurogenesis, actin, nestin, striatum, thalamus

## Abstract

Previously we demonstrated, in rats, that treatment with growth hormone (GH) and rehabilitation, carried out immediately after a motor cortical ablation, significantly improved the motor affectation produced by the lesion and induced the re-expression of nestin in the contralateral motor cortex. Here we analyze cortical proliferation after ablation of the frontal motor cortex and investigate the re-expression of nestin in the contralateral motor cortex and the role of the striatum and thalamus in motor recovery. The rats were subjected to ablation of the frontal motor cortex in the dominant hemisphere or sham-operated and immediately treated with GH or the vehicle (V), for five days. At 1 dpi (days post-injury), all rats received daily injections (for four days) of bromodeoxyuridine and five rats were sacrificed at 5 dpi. The other 15 rats (*n* = 5/group) underwent rehabilitation and were sacrificed at 25 dpi. GH induced the greatest number of proliferating cells in the perilesional cortex. GH and rehabilitation produced the functional recovery of the motor lesion and increased the expression of nestin in the striatum. In the thalamic ventral nucleus ipsilateral to the lesion, cells positive for nestin and actin were detected, but this was independent on GH. Our data suggest that GH-induced striatal nestin is involved in motor recovery.

## 1. Introduction

Traumatic brain injury (TBI) represents one of the biggest health problems in developed countries, both because of the number of deaths it causes as well as the high percentage of those affected who suffer from some type of motor and/or cognitive disability as a result of the injury. TBI directly affects the meningeal or brain cranial structures, causing an alteration in neurological function due to the damage suffered by neurons, glial cells, and blood vessels located in the damaged area. A major problem in the treatment of patients who survive a TBI is the poor endogenous potential for recovery that the brain has, due to the lack of axonal regeneration, mainly produced by the acute expression of Nogo-A, an inhibitor of axonal outgrowth, and the limited ability to replace damaged neuronal cells [1]. However, some patients with TBI or stroke show spontaneous and gradual improvement during the first 6–12 months after the injury [2]. This improvement is attributed to structural and functional compensatory changes that occur in the damaged brain, carried out by what is described as neuroplastic mechanisms. The possibility of acting by enhancing these mechanisms, both pharmacologically and through rehabilitation therapies, opens up new perspectives in the recovery of patients with TBI [3]. In fact, in the undamaged brains of adult rats, the learning of new tasks induces increases in dendritic spines turnover [4], and focal cortical lesions induce cortical plasticity [5,6], new axonal connections [6,7] and neurogenesis [8]. However, while there is already evidence indicating that rehabilitation can increase the number of dendritic spines and their complexity in the undamaged hemisphere of adult rats [9,10,11,12], the opposite situation seems to occur in the perilesional cortex, at least after a stroke [13,14,15]. While it is clear that rehabilitation plays an important role in recovery from neurological injury, its effects are strongly increased when it is accompanied by medical treatments, as previously suggested [3]. One of these treatments consists of the administration of growth hormone (GH), a hormone that, together with its mediator insulin-like growth factor (IGF-I), plays a key role in fetal neurodevelopment [16,17], but also significantly contributes to enhancing the effects of rehabilitation after a brain injury in animal models [18,19,20,21,22,23,24,25,26] and human patients [27,28,29,30,31,32,33,34].

In previous studies, in rats, we demonstrated that GH administration, given immediately or very soon after a severe ablation of the frontal motor cortex of the dominant hemisphere, induced a marked improvement of motor skills in the paw-reaching-for food task, while rats treated with vehicle did not change their performance despite intense rehabilitation [35,36]. We attributed these results to the appearance of greater brain plasticity in the contralateral motor area, observing re-expression of nestin [35], or nestin and actin [36], in this intact area, most likely because compensatory brain plasticity had been established, leading to a marked improvement in the functionality of the paw affected by the lesion. Given that nestin is a class VI intermediate filament protein expressed in uncommitted neural progenitor cells (NPCs) [37,38,39,40], it could be thought that the results obtained had been produced by GH-induced adult neurogenesis; however, in our previous study [35], nestin immunoreactivity (ir) was detected in the synaptic terminals outlining neuronal bodies, suggesting that these nestin-expressing neurons were mature neurons and began to re-express nestin after ablation of the frontal motor cortex and treatment with GH and rehabilitation. Therefore, the objective of this new study was to investigate cortical proliferation after ablation of the motor cortex to elucidate whether this re-expression of nestin was due to neurogenesis or neuronal plasticity mechanisms that occur in the motor cortex of the undamaged hemisphere, as well as to analyze the role of subcortical areas involved in motor activity, such as the striatum and the thalamus, in motor recovery induced by the administration of GH and rehabilitation after cortical ablation.

This study was conducted in 20 adult male Wistar rats (Charles River Laboratories, Barcelona, Spain), weighing 200–220 g at the beginning of the experiments. Animals were housed under conditions of controlled temperature (18–20 °C) and natural light/dark cycle; they were allowed to acclimatize to the animal facilities for several days after arrival, before starting the experiments. They were fed with a normal chow diet and water *ad libitum*, except when the paw-reaching-for-food task was performed. During this time, animals were moderately food restricted until their body weight was reduced to 86–88% of its initial *ad libitum* weight. 

## 2. Results

### 2.1. Treatment with GH Immediately after Lesion plus Rehabilitative Therapy Produced the Functional Recovery of the Motor Impairment Induced by the Cortical Ablation

**Figure 1:**
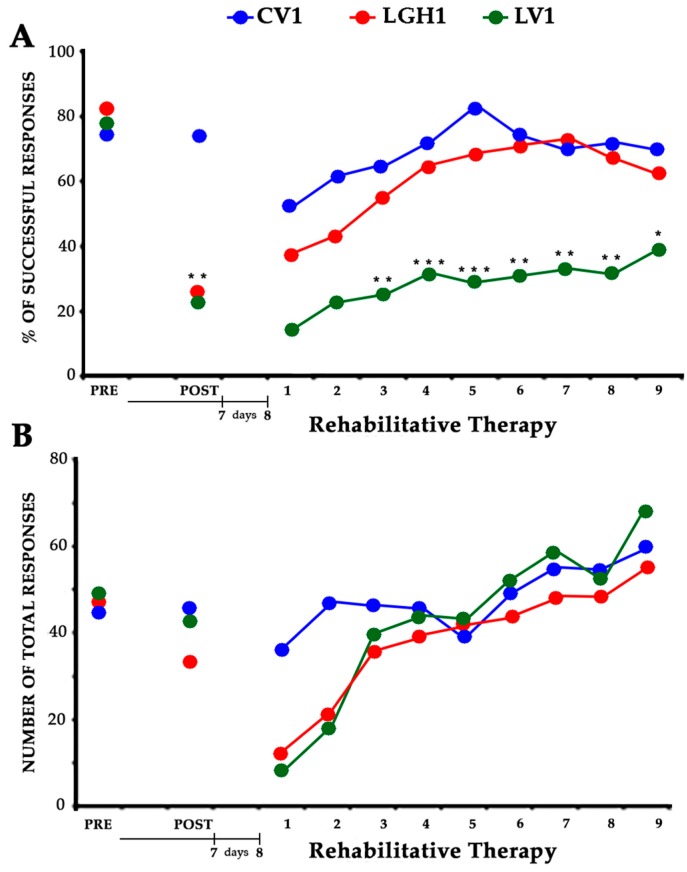
Effectiveness of the treatment with GH and rehabilitation. Animals treated with GH (LGH1, red graph) or vehicle (LV1, green graph) immediately after cortical ablation. Results obtained in the paw-reaching-for-food task with the preferred paw (impaired paw) at the presurgical phase, (PRE), 7 dpi (days post-injury) (POST) and rehabilitative therapy (at 8 dpi). (**A**) Mean percentage of successful responses (successful responses/total number of responses). (**B**) Mean of the total number of responses (successful plus unsuccessful with both paws). The rehabilitative therapy consisted in the obliged use of the impaired paw, in daily sessions of 3 min for nine consecutive days. Significance levels are done with respect to the sham-operated control group (CV1, blue graph). *** *p* < 0.001; ** *p* < 0.005; * *p* < 0.01 (Bonferroni test). The x-axis indicates days.

In the presurgical phase, the ability of taking food pellets from the groove and eating them was similar in all rats. All rats showed a similar strategy, using a single forelimb to reach the pellets; this allowed us to establish the spontaneous limb preference during training. In all animals, the limb preference was established by the fourth session, and the paw used was considered the preferred paw. When the percentage of attempts with the right or left paw was between 85% and 100%, a rat was classified as right-handed or left-handed. The animals were considered well trained when the percentage of successful responses was ≥60% during two consecutive sessions. To distribute the animals in the different experimental groups, the average of the results obtained in the last two sessions of this phase was taken. Therefore, as Figure 1 shows, both the percentage of successful responses (A) and the total number of responses (B) were similar in all experimental groups (Figure 1A PRE, F_2,12_ = 0.477; Figure 1B PRE, F_2,12_ = 0.140).

The effectiveness of the cortical ablation was tested at 7 dpi (days post-injury). Frontal motor cortical ablation was very important and homogeneous (size and location), as in other studies from our group [35,36,41]. Lesions were restricted to the primary (M1) and secondary (M2) motor cortex areas. ANOVA indicated significant differences between groups (F_2,12_ = 8.956, *p* ≤ 0.01). The percentage of successful responses was maintained in the control group (CV1), but significantly decreased in all animals with cortical ablation (Bonferroni post hoc test, *p* ≤ 0.0001), (Figure 1A POST). Some of these lesion animals changed their preferred paw (disuse of the paw contralateral to the lesion and proportionately increased reliance on the paw ipsilateral to the lesion), while others continued to use the preferred paw, although in this case the number of successful responses clearly decreased. Regarding the total number of responses, the global ANOVA did not show significant differences between groups (Figure 1B POST).

All animals underwent rehabilitation therapy, beginning at 8 dpi, in daily sessions of 3 min, for nine consecutive days (see the experimental design in Section 4). The rehabilitation consisted in the forced use of the impaired paw (preferred paw) by means of a bracelet fitted on the nonpreferred paw. Two-way (group and session) ANOVA showed significant differences between groups (F_2,12_ = 15.52; *p* ≤ 0.0005). In all groups the number of successful responses was increasing throughout the rehabilitation sessions, therefore a significant session effect was found (F_8,96_ = 4.85, *p* ≤ 0.0001). Conversely, the interaction group x session was not significant (F_16,96_ = 0.473). The partial ANOVA tests indicated significant differences between groups from the fourth to the ninth sessions of rehabilitation. However, the Bonferroni post hoc test indicated that the percentage of successful responses throughout the sessions clearly increased in animals treated with GH immediately after the cortical ablation (LGH1). As Figure 1A shows, the performance in the paw reaching test of the animals treated with GH was similar to that of the animals of the control group throughout the nine rehabilitation sessions (Figure 1A). This significant improvement was not observed in lesion animals treated with vehicle (LV1); in them, a lower percentage of successful responses, statistically significant, was observed as compared to control animals or animals treated with GH (Figure 1A; significance levels are done with respect to the sham control group, CV1). The total number of responses was similar in all the experimental groups; therefore, no significant differences were observed among them (F_2,12_ = 0.908) (Figure 1B). However, since all groups progressively increased the number of total responses throughout the rehabilitative sessions a marked session-effect (F_8,96_ = 17.69, *p* ≤ 0.0001) existed. The interaction of group x session was not significant (F_16,96_ = 1.603).

### 2.2. Cortical Cell Proliferative Response in Animals Treated with GH or Vehicle after Motor Cortical Ablation

**Figure 2:**
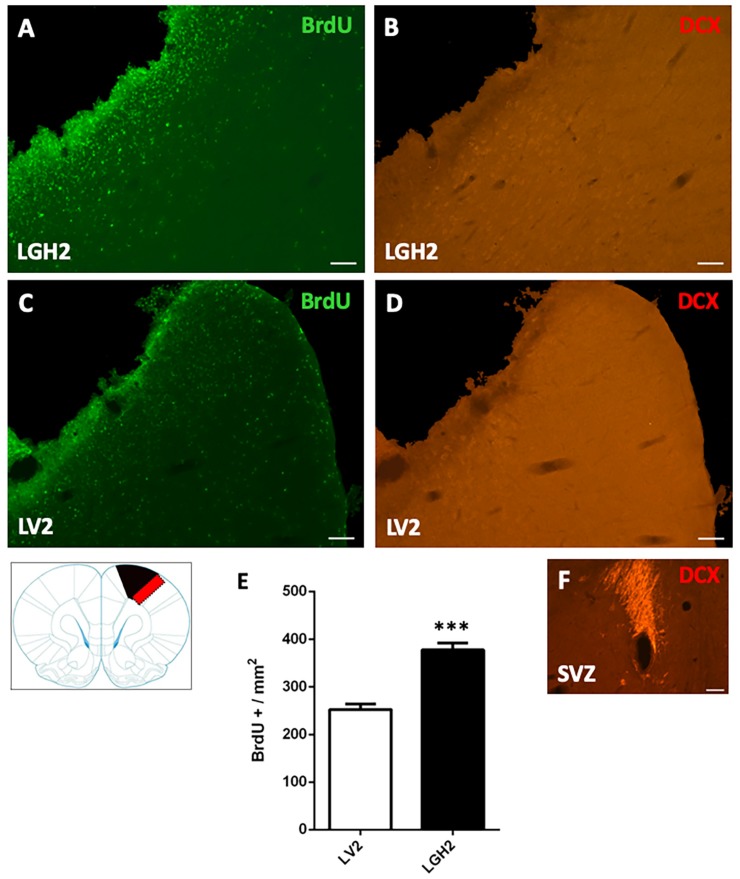
Cell proliferation in the perilesional motor cortex of animals sacrificed at 5 dpi. BrdU and doublecortin (DCX) labeling in the perilesional primary motor cortex, M1 (red rectangle in the scheme) of animals treated with GH (**A**,**B**) or vehicle (**C**,**D**), immediately after cortical ablation and sacrificed at 5 dpi. Coronal sections at +2.52 mm from Bregma. Scale bar = 200 µm. In (**E**) is shown the significant increase in BrdU-labeled cells (mean + SEM, *n* = 2) observed in GH-treated animals compared to vehicle-treated animals. (**F**) positive control of the DCX labeling in the subventricular zone (SVZ) of the lateral ventricle. In the scheme, the black image indicates the place where the ablation was produced. *** *p* < 0.0001 (Student’s *t*-test).

To determine the cortical cell proliferative response in animals treated with GH or vehicle after motor cortical ablation, rats were injected with the mitotic marker bromodeoxyuridine (BrdU). In animals with cortical ablation treated with GH and sacrificed at 5 dpi, a large number of BrdU+ cells in the perilesional motor cortex was detected. This BrdU labeling was significantly higher in animals treated with GH than in animals treated with vehicle (*p* < 0.0001) (Figure 2A,C,E). However, in the perilesional motor cortex of animals treated with GH or vehicle, no doublecortin (DCX) labeling, a marker of proliferating neuroblasts, was observed (Figure 2B,D).

**Figure 3:**
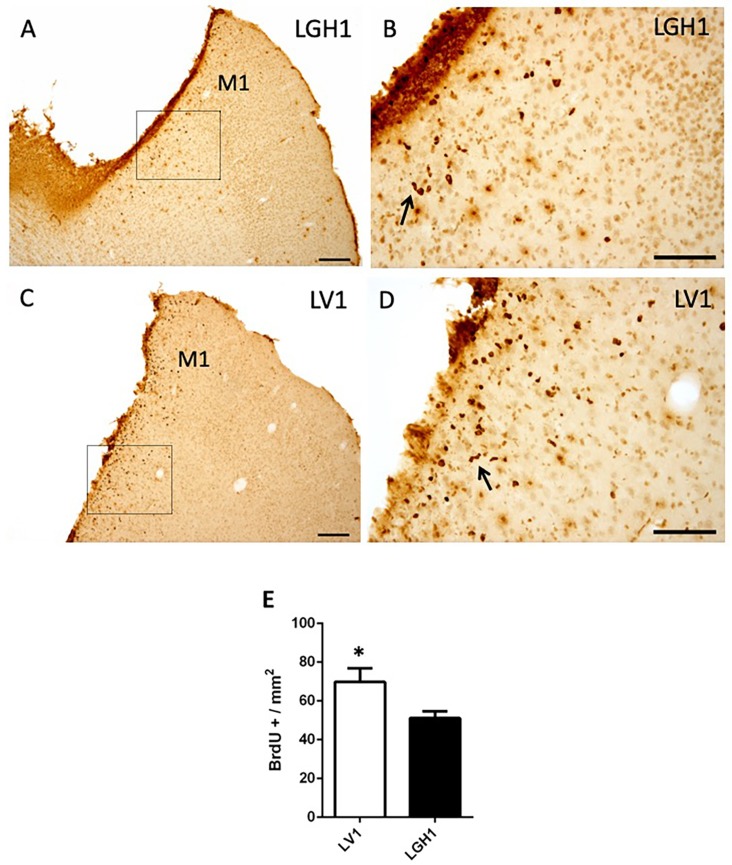
Cell proliferation in the perilesional motor cortex of animals sacrificed at 25 dpi. BrdU immunolabeling in the perilesional primary motor cortex (M1) of animals treated with GH (**A**,**B**) or vehicle (**C**,**D**), immediately after cortical ablation and sacrificed at 25 dpi. (**B**,**D**) are a magnification of (**A**,**C)**, respectively. Coronal sections at +2.52 mm from Bregma. The black arrows in B and D point out BrdU+ cells forming clusters (or chains). Scale bars: 200 µm (**A**,**C**), 100 µm (**B**,**D**). (**E**) Quantification of the total number of BrdU+ cells in the perilesional M1 motor cortex of animals treated with GH or vehicle. A significant increase in BrdU-labeled cells in animals treated with vehicle compared with animals treated with GH was found (Mean + SEM; *n* = 2). * *p* < 0.05.

To evaluate the functional benefits of the GH treatment, in the other group of animals treated with GH or vehicle that were sacrificed at 25 dpi, a notably decrease of proliferating cells (BrdU+) in the perilesional motor cortex was observed (Figure 3), in comparison to animals sacrificed at 5 dpi (Figure 2). Furthermore, a smaller number of BrdU+ cells were detected in the perilesional motor cortex of animals treated with GH (Figure 3A,B) as compared to animals treated with vehicle (Figure 3C,D); however, although statistically significant, the magnitude of the differences was small (*p* < 0.05). Some BrdU+ cells showed a bigger size, probably corresponding to reactive astrocytes and some formed clusters or chains (Figure 3B,D). BrdU+ cells were also detected, scattered in the adjacent somatosensorial cortex and in the corpus callosum (Figure 3A). In control animals, some few BrdU+ cells were observed in the cortex underlying the region where the sham operation was performed, probably due to slight damage in that area, suggesting that sham operation was similar to a mild lesion. 

**Figure 4:**
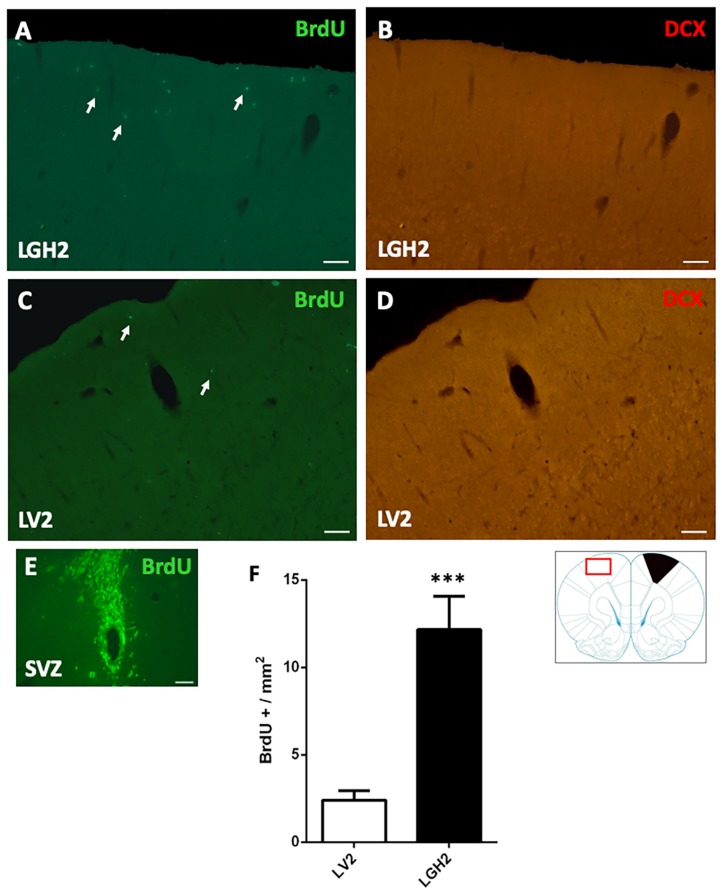
Cell proliferation in the contralateral motor cortex of animals sacrificed at 5 dpi. BrdU and doublecortin (DCX) labeling in the contralateral motor cortex (red rectangle in the scheme) of animals treated with GH (**A**,**B**) or vehicle (**C**,**D**), immediately after cortical ablation and sacrificed at 5 dpi. (**E**) positive control of the BrdU labeling in the subventricular zone (SVZ) of the lateral ventricle. Arrows point to some BrdU+ cells. Coronal sections at +2.52 mm from Bregma. Scale bar = 200 µm. (**F**) shows the significant increase in BrdU-labeled cells (mean ± SEM, *n* = 2) observed in GH-treated animals compared to vehicle-treated animals. The black region indicates where the ablation was produced. *** *p* < 0.001 (Student’s *t*-test).

In animals treated with GH and sacrificed at 5 dpi, the contralateral motor cortex showed some BrdU+ cells dispersed in the motor cortex (Figure 4A). In animals treated with vehicle, a notably decrease of BrdU+ cells in the motor cortex, in comparison with animals treated with GH, was observed (Figure 4C). This BrdU labeling was significant higher in animals treated with GH than in animals treated with vehicle (*p* < 0.001) (Figure 4A,C,F). However, in the contralateral motor cortex of animals treated with GH or vehicle, no doublecortin (DCX) labeling was detected (Figure 4B,D).

**Figure 5:**
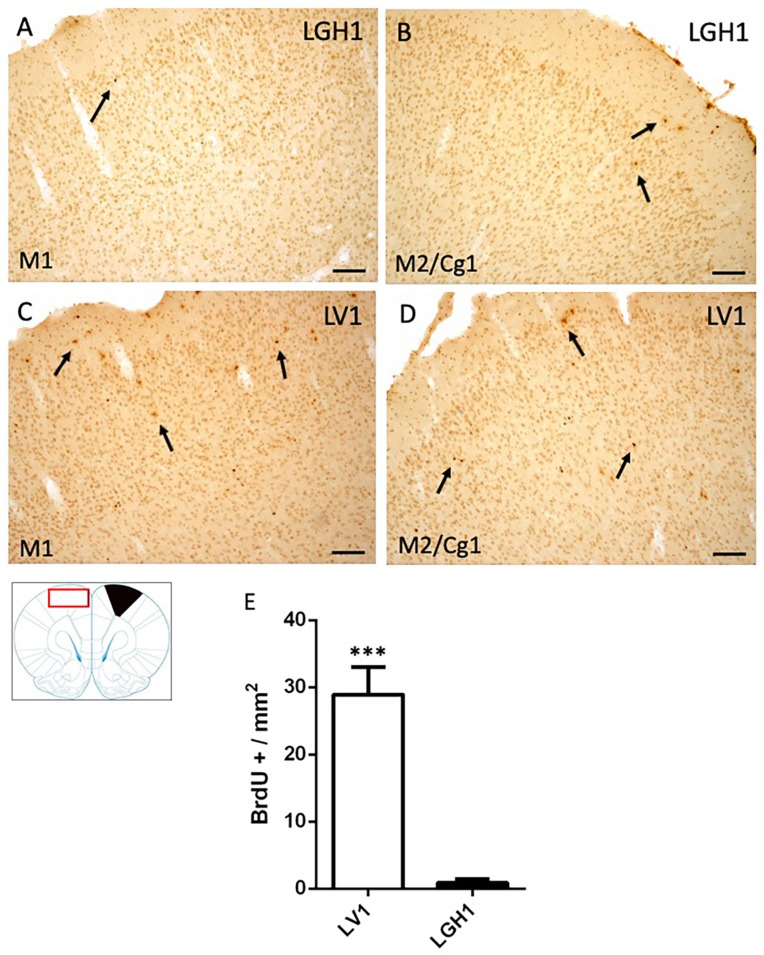
Cell proliferation in the contralateral motor cortex of animals sacrificed at 25 dpi. BrdU-ir in the contralateral motor cortex (red rectangle in the scheme) of animals treated with GH (**B**) or vehicle (**C**,**D**) at 25 dpi. (**A**,**C**), primary motor cortex (M1); (**B**,**D**), secondary motor cortex/cingulate cortex, area 1, (M2/Cg1). Arrows point to some BrdU+ cells. Coronal sections at +2.52 mm from Bregma. The black region indicates where the ablation was produced. Scale bar = 100 µm. (**E**) shows the significant increase in BrdU-labeled cells (mean + SEM, *n* = 2) observed in vehicle-treated animals compared to animals treated with GH *** *p* < 0.001 (Student’s *t*-test).

In the contralateral motor cortex of animals treated with GH, some few BrdU+ cells dispersed in the M1 cortex were detected at 25 dpi (Figure 5A). In the secondary motor and cingulate cortices (M2/Cg1), a slight increase of BrdU+ cells was observed, mainly in layer I (Figure 5B). These BrdU immunostaining was similar to that found in control animals. In animals treated with vehicle, a significant increase of BrdU^+^ cells in M1 and M2/Cg1 cortices, in comparison with animals treated with GH, was detected (Figure 5C–E).

### 2.3. Nestin Expression in the Striatum and Thalamus of Animals Treated with GH or Vehicle after Cortical Ablation

**Figure 6:**
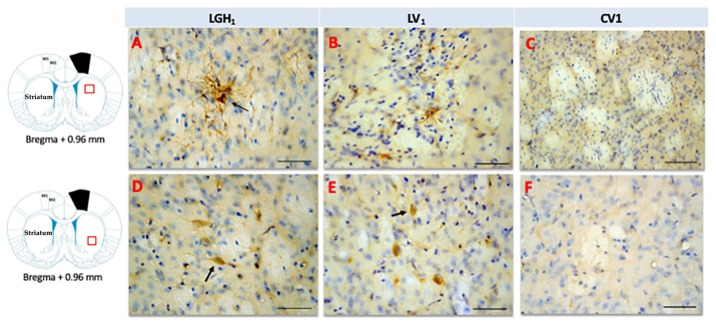
Nestin expression in the striatum ipsilateral to the lesion of animals sacrificed at 25 dpi. Nestin re-expression in the striatum ipsilateral to the lesion of animals belonging to the three experimental groups LGH1 (**A**,**D**), LV1 (**B**,**E**) and CV1 (**C**,**F**). The three microphotographs to the right correspond, in each experimental group, to the red box indicated in the scheme. Coronal sections at +0.96 mm from Bregma. The arrow in (**A**) points to a reactive astrocyte at the striatal white matter. The arrows in (**D**,**E**) point to nestin+ neurons in the striatal gray matter. The re-expression of nestin in the striatum was observed from AP = +2.52 mm to AP = −0.12 mm with respect to bregma. Scale bar = 100 µm. In the scheme, the black trapezoid indicates the place where the lesion was produced.

In the striatum of animals treated with GH, nestin expression was detected in the striatal white matter, mainly ipsilateral to the lesion; highlighting the presence of cells nestin+ with a morphology similar to reactive astrocytes and fibrillar structures. Therefore, it is likely that nestin immunoreactivity (ir) corresponds to astrocytic prolongations (Figure 6A). This nestin immunostaining in the striatal white matter was observed in the dorsal striatum (at +2.4 mm from Bregma) and, at more caudal sections (from Bregma +1.08 mm to Bregma −0.12 mm) in the medial-central striatum. This nestin expression at the striatal white matter, ipsilateral to the lesion, was markedly lower in animals treated with vehicle (Figure 6B). In control animals, nestin expression was restricted to blood vessels (Figure 6C). At the medial/ventral striatum, ipsilateral to the lesion, of animals treated with GH or vehicle, some cells nestin+ with a neuronal morphology, scattered in the striatal gray matter, were detected (Figure 6D,E). In controls, at the medial/ventral striatum, nestin expression was restricted to blood vessels and occasionally was seen some few cells weakly nestin+ with a neuronal morphology (Figure 6F). In the striatum contralateral to the lesion, occasionally some few fibrillar structures nestin^+^ located at the striatal white matter, probably corresponding to astrocytic prolongations, were detected; in addition, at the medial/ventral striatum, some few cells nestin^+^ with neuronal morphology were observed. 

**Figure 7:**
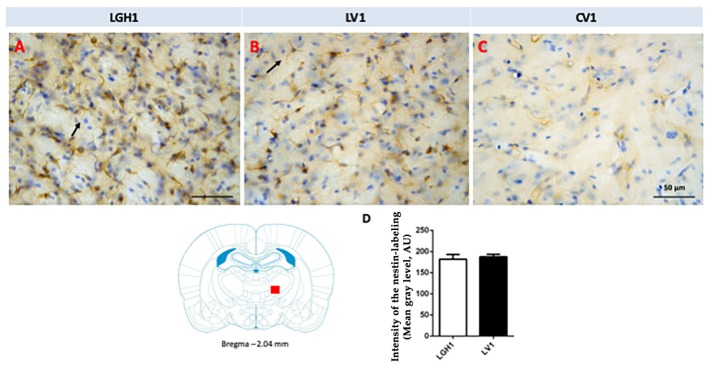
Nestin expression in the ventral thalamic nucleus, ipsilateral to the lesion, of animals belonging to the three experimental groups (LGH1, LV1 and CV1), sacrificed at 25 dpi. (**A**) Nestin-ir fibers in the ventral thalamic nucleus of a rat treated with GH immediately after the lesion can be seen. (**B**) A similar nestin immunolabeling can be seen in a rat treated with vehicle immediately after the injury was produced. (**C**) In sham-operated animals, the expression of nestin is restricted to blood vessels. The arrows point to the microglia prolongations. (**D**) Mean nestin reaction intensity of gray level variation expressed in arbitrary units (AU); values represent mean + SEM, *n* = 2. No differences were found in the intensity of nestin reaction. Coronal sections at −2.04 mm from Bregma. The red square shows where the upper photomicrographs correspond. Scale bar: 50 µm.

In the thalamus of animals treated with GH (LGH1 group) or vehicle (LV1 group), nestin expression was detected in the thalamic ventral nucleus, ipsilateral to the lesion (ventrolateral, VL, and anterior ventral, VA, nuclei). This expression of nestin was observed in fibrillar structures, probably corresponding to prolongations of microglia cells, being similar in groups LGH1 and LV1 (Figure 7A,B,D). Conversely, no nestin-ir was observed in the thalamus contralateral to the lesion. In controls, the expression of nestin was restricted to blood vessels (Figure 7C).

### 2.4. Actin Expression in the Striatum and Thalamus of Animals Treated with GH or Vehicle after Cortical Ablation

**Figure 8:**
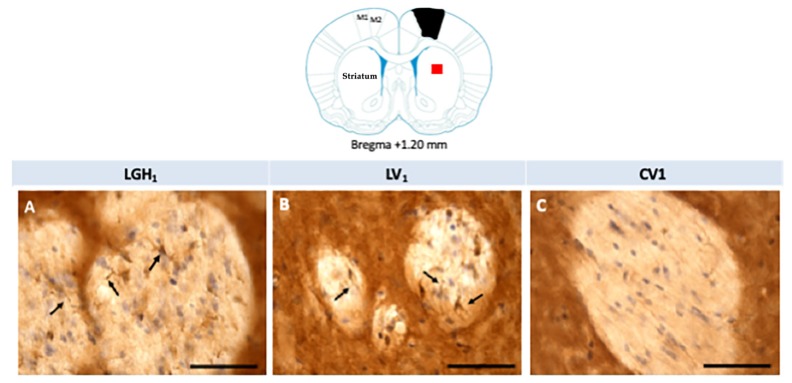
Actin immunoreactivity in the striatal white matter, ipsilateral to the lesion. Fine fibers, actin+, were observed in a rat treated with GH (**A**) or vehicle (**B**). However, no actin immunostaining was found in sham-operated control animals (**C**). Arrows point to actin+ fibers. Coronal sections at +1.20 mm from Bregma. The red square indicates where the images were taken. The black trapezoid indicates where the ablation of the motor frontal cortex was done. Scale bar = 50 µm.

Actin expression was detected in the striatum, ipsilateral to the lesion, of animals treated with GH (LGH1) or vehicle (LV1), but this expression of actin was similar in both groups, as Figure 8 shows. Fine fibers, actin+, were observed in the striatal white matter of rats treated with GH (LGH1) or vehicle (LV1) (Figure 8A,B). In control animals (CV1), no actin immunostaining was observed in the striatum (Figure 8C). No actin expression was observed in the striatum contralateral to the lesion in any of the groups studied. 

**Figure 9:**
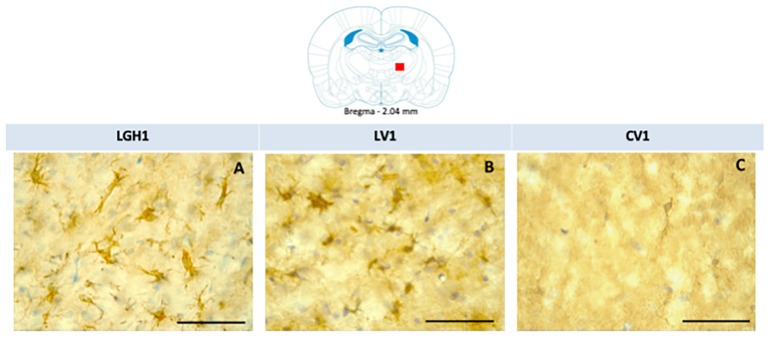
Actin immunoreactivity in the thalamic ventral nucleus (VA/VL complex) ipsilateral to the lesion in the three experimental groups of animals. Active microglia/macrophage-like cells, actin+, in a rat treated with GH (**A**) or vehicle (**B**). No differences were observed in the actin-immunoreactivity (ir) between animals treated with GH (**A**) or vehicle (**B**) immediately after injury. No actin-ir was found in control animals (**C**). Scale bar: 50 µm. Coronal sections at −2.04 mm from Bregma. The red square indicates the place where the images were taken.

**Figure 10:**
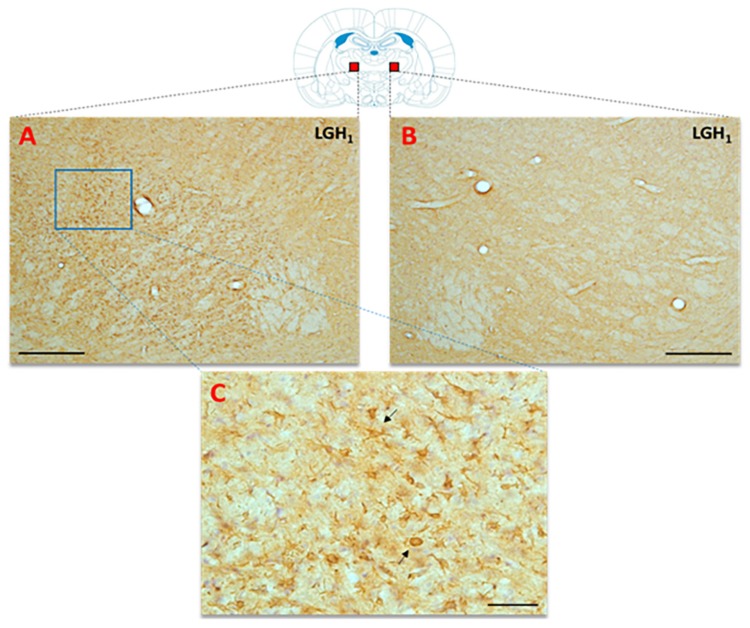
Actin immunoreactivity in the thalamic ventral nucleus (VA/VL complex) ipsilateral to the lesion, from an animal treated with GH. Actin-ir was detected in the thalamic ventral nucleus ipsilateral to the lesion in animals treated with GH (**A**), or vehicle (not shown in this image), immediately after injury, but not in the contralateral thalamic ventral nucleus (**B**). (**C**) Magnification of the blue rectangle shown in (**A**); the black arrows indicate some actin+ cells exhibiting an active microglia/macrophage-like morphology. Scale bars: 200 µm in (**A**,**B**), and 50 µm in (**C**). Coronal sections at −1.92 mm from Bregma. Red squares indicate the place where the images were taken.

In the thalamus of animals treated with GH or vehicle, actin expression was detected in the ventral nucleus (VL and VA nuclei), ipsilateral to the lesion (Figure 9), but not in the contralateral hemisphere (Figure 10). Actin expression was located in cells of poorly defined limits, with a robust structure and with few short and irregular prolongations; the morphological characteristics were similar to activated microglia/macrophage cells (Figure 9A,B and Figure 10C). No actin immunoreactivity was detected in controls (Figure 9C).

## 3. Discussion

Our results confirm previous studies from our group showing that the administration of GH immediately after a cortical ablation together with rehabilitation induces the functional recovery of the deficit in the manual ability caused by the injury, in adult rats [35,36]. This recovery depends on both GH and rehabilitation since it was observed when the use of the preferred hand was forced (the hand affected by the injury) as soon as the rehabilitative therapy started, as Figure 1A shows. Our results also indicate that the ablation of the frontal motor cortex induced cell proliferation in the perilesional injured cortical area in the rats sacrificed at 5 dpi, an effect significantly enhanced by GH; this is in concordance with the findings that we observed in the hippocampus of rats in which we induced damage with kainic acid, and GH administration increased the number of newly formed neural precursors [24]. However, this marked cortical proliferation was not translated into the appearance of neuroblasts, since we did not detect DCX labeling in the perilesional cortical area, and DCX is a marker of cells restricted to neuronal lineage or neurons [42], although it has been described that after an injury the neuroblasts migrate from the subventricular zone (SVZ) to the site of the injury [43]. Most likely, the absence of detection of DCX labeling in our study is related to the time of the sacrifice after the injury, because it has been shown that DCX+ cells are present in the perilesional area up to only 3 dpi [44]. Therefore, the possibility that GH induced adult neurogenesis for repairing the injured area, as we previously postulated [35,36], cannot be excluded, although the magnitude of the lesion produced would impede any possibility of significant functional improvement (repair mechanisms may be different depending on the type of injury, its severity and the region of the brain). Moreover, it has been demonstrated that the cell proliferation observed in the perilesional cortex most likely corresponds to microglial cells and astrocytes [44]. At 25 dpi, the cell proliferation in the perilesional area was considerably lesser than that observed at 5 dpi, which is logical since BrdU decreases as cell division occurs.

We previously reported that there was a re-expression of nestin in the motor cortex contralateral to the lesion in animals treated with GH immediately after the frontal motor cortex ablation, which repaired the motor deficit [35]. This was the reason why in this study we tried to investigate whether this re-expression of nestin in the contralateral cortex occurred in newly formed cells or if it was produced in resident cells. The fact that in the current study we observed few cells BrdU+ scattered in the contralateral frontal motor cortex of GH-treated animals suggest that the re-expression of nestin in this zone would be due to mechanisms of neural plasticity in resident cells and not to neurogenesis. However, although it is not very plausible, we cannot completely rule out that the different moment of the animals’ sacrifice after the injury (at 25 dpi in the present study and at eight weeks in the previous study) could have affected the results obtained.

Interestingly, we found expression of nestin in the striatum of both hemispheres of rats treated with GH and rehabilitation after the frontal motor cortex ablation, being much more marked in the ipsilateral striatum. It is well known that all areas of the cortex send excitatory glutamatergic signals to specific areas of the striatum, which also receives excitatory signals from the thalamus. The motor cortex projects mainly to the ipsilateral striatum, although it also sends projections, to a lesser extent, to the striatum of the contralateral hemisphere. Electrophysiological methods have demonstrated that the same cortical projection neuron sends its main axon to the ipsilateral striatum, but this neuron also sends a collateral branch, through the corpus callosum, which bifurcates into two branches, one of which is directed towards the homotopic contralateral cortex and the other towards the homotopic contralateral striatum [45]. In motor aspiration injuries, it has been shown that cortical neurons of the intact motor cortex not only project to the ipsilateral striatum, but also, with a minor cross-component, to the contralateral striatum to the lesion [46]. In addition, another study shows that cortical lesions cause reactive gliosis in the striatum, accompanied by a structural reorganization of the dendritic axis of striatal target neurons, as well as a replacement in the synapses by axons of the contralateral cortex [47]. All this would explain the expression of nestin observed in this study, evidenced by the labeling of reactive astrocytes, astrocyte prolongations, and neurons, in both the ipsilateral and contralateral striatum to the lesion. We found nestin+ neurons in the ventral area of the striatum in injured animals, both in those treated with GH and with the vehicle, which seems to indicate that nestin can exert neurotrophic functions after brain injury, as previously suggested [48] (in the control animals we also found a few weakly marked neurons in the ventral striatum). Moreover, we found that actin was expressed in very fine fibrillar structures, which possibly correspond to astrocyte prolongations, in the white substance of the ipsilateral striatum to the lesion; this, together with the expression of nestin in reactive astrocytes, in astrocyte prolongations and in neurons, in the ipsilateral and contralateral striatum to the lesion, suggests that nestin and actin could be involved in the neuroanatomic remodeling that the corticostriatal pathway undergoes after a cortical lesion by aspiration [46]. It is possible that the expression of actin and nestin in the striatum ipsilateral to the lesion, in animals with cortical ablation, reveals cortical-efferent plasticity mechanisms that occur in the striatum due to the lack of cortical afferents. It has been suggested that a loss of afferent signals may lead to glial activation, an increase in trophic factors or changes in local substrate molecules that affect neuronal growth [49], which may constitute a potential strategy to improve cortical-efferent connectivity after cortical damage [46]. On the other hand, the greater expression of nestin in the striatum of animals treated with GH that functionally recovered the motor deficit caused by the injury would suggest a role of nestin in the benefits of treatment with GH and rehabilitation that would have led to the recovery of the motor deficit. The positive effects of GH on functional recovery after brain injury have already been described in many studies [18,19,20,21,22,23,24,25,26,27,28,29,30,31,32,33,34,35,36], although the exact mechanisms involved in the positive role played by GH have not yet been fully clarified.

Interestingly, we observed that after cortical ablation there was a marked expression of actin and nestin in the anterior ventral (VA) and ventrolateral (VL) thalamic nuclei ipsilateral to the lesion, both in rats treated with GH and in those who received vehicle. In rats, VA and VL are generally indistinguishable on cytoarchitectural grounds [50,51], and are often treated as a single nucleus, the VA/VL complex, with topographic thalamocortical projections associated with the motor cortex [52]. After brain damage or sensory deprivation, thalamocortical projections can exhibit considerable reorganization in terms of increased neural plasticity [53].

In our study, actin labeling in the VA/VL complex was located in cells with poorly defined boundaries and few branches, a morphology similar to activated microglia/macrophage cells. It is known that the glial response to an injury involves the activation of microglia cells, together with a marked astroglial reactivity [54]. Microglial activation and proliferation are features of traumatic injury to the central nervous system [55]. Microglia/macrophage cells are able to change the length and thickness of their prolongations after an injury, and it has been suggested that actin may play a determining role in the morphological changes that underlie the phenotypic modifications of the microglia after an injury [56]. On these bases, it seems clear that the detection in our study of activated microglia/macrophage cells, actin+, in the VA/VL complex ipsilateral to the lesion, would have occurred as a consequence of the cortical ablation performed.

The expression of nestin in the VA/VL complex, ipsilateral to the lesion, was also notable, although the nestin labeling was morphologically distinct from that obtained for actin. Nestin expression occurred in nestin+ fibrillar structures, most likely corresponding to microglia cell extensions. Since nestin is a protein of intermediate class VI filaments, which are located in the cytoplasm of the cell, while actin constitutes microfilaments that are located in the proximity of the cytoplasmic membrane giving mechanical support to the cell membrane, due to its binding to it by means of membrane anchoring proteins, it is not surprising that the morphology of the actin labeling in the microglia cells does not coincide with the nestin labeling.

In any case, the expression of nestin and actin in activated microglia/macrophage cells, in the VA/ VL complex ipsilateral to the lesion, has to be due to cortical ablation, since it was observed in both injured animals treated with GH and in those treated with vehicle, indicating that the expression of these proteins is involved in the compensation mechanisms that appear after an injury to try to compensate for the damage.

In summary, in this study we demonstrated that the treatment with GH, administered immediately after the frontal motor cortex ablation, and rehabilitation, induced a functional recovery of the deficit in manual ability caused by the lesion in adult rats; we also observed that this ablation stimulated cell proliferation in the perilesional cortex and this effect was significantly enhanced by GH treatment in animals sacrificed at 5 dpi, although it decreased markedly at 25 dpi. Our data also indicate that the expression of nestin in the contralateral motor cortex in animals treated with GH that recover from their motor deficit would depend on mechanisms of neural plasticity in resident cortical cells and not on *de novo* neurogenesis. We also found an increase in the expression of nestin in the striatum of GH-treated rats that could be involved in the functional recovery observed in these animals after cortical ablation. This injury also induced the expression of nestin and actin in activated microglia/macrophage cells, in the VA/VL complex ipsilateral to the lesion, but this was not due to any GH effect.

## 4. Materials and Methods

### 4.1. Experimental Design and Behavioral Test

All procedures were approved (BIO/SA64/14, 13 November 2014) by the University of Salamanca Ethics Committee and were conducted in accordance with the animal care guidelines of the European Communities Council (2010/63/UE) and Spanish normative (RD 53/2013 and law 32/2007); efforts were made to minimize suffering and the number of animals used.

**Figure 11:**
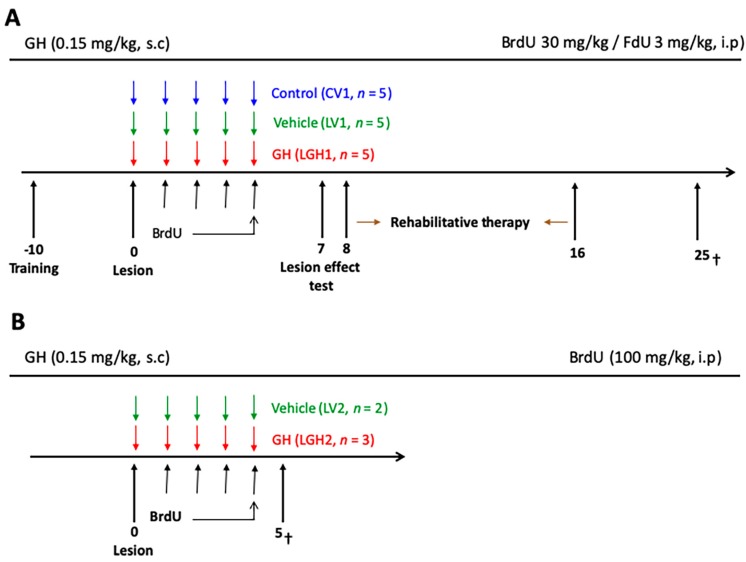
Schematic diagram of the experimental design. (**A**) The treatment with GH or vehicle started immediately after the lesion (LGH1 and LV1 groups, respectively). Sham-operated controls were treated with vehicle (CV1). The injections of BrdU/FdU commenced on day 1. On day 7 after lesion, the effectiveness of the cortical ablation was evaluated in the paw reaching test. The rehabilitative therapy consisted in daily sessions of 3 min, with the obliged use of the impaired paw, for nine consecutive days. Animals were sacrificed at day 25 after lesion. (**B**) Diagram of the experimental design of animals treated with GH or vehicle and sacrificed at day 5 after injury. Day 0 indicates the day of the cortical ablation. Time scale days.

The experimental design consisted of the following phases: (i) presurgical behavioral test; (ii) ablation of the frontal cortex; (iii) treatment with vehicle (LV1 and control group CV1) or GH (LGH1), subcutaneously (s.c), immediately after the cortical ablation, and intraperitoneal (ip) BrdU/FdU administration, followed by rehabilitative therapy beginning at 8 dpi and carried out for nine days; these study phases are schematically represented in Figure 11A. Surgical procedures and sacrifice were carried out under deep anesthesia with Equithesin (Chloral hydrate/magnesium sulfate/pentobarbital sodium; 20 mg/kg, ip,). The animals were sacrificed 25 days post-injury. Other animals (*n* = 5) received likewise vehicle (LV2, *n* = 2) or GH (LGH2, *n* = 3) immediately after the lesion, and BrdU, but they were sacrificed at 5 dpi (Figure 11B), without receiving rehabilitative therapy.

**Figure 12:**
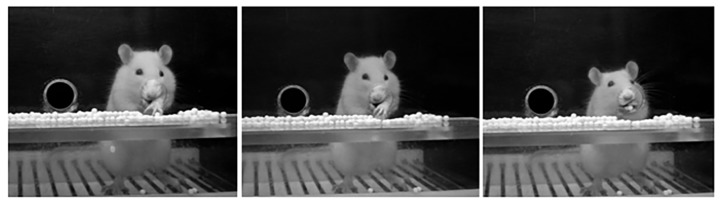
Paw-reaching-for-food task in the test cage. Three consecutive photographs showing a rat in the test cage performing a successful response in the paw-reaching test during the training in the pre-surgical phase. The design of the test cage prevents the use of the tongue to retrieve food pellets or to rake the pellets.

Eight days after the arrival of animals they were trained for the paw-reaching-for-food task, a specific motor test for fine motor skills that we used in our previous studies [35,36,41]. In this test, carried out in a special cage, as described in a previous study from our group [41], animals are conditioned to perform high-precision motor movements of extension and flexion of the forelimb fingers for obtaining food. Before carrying out the test, animals were housed individually and, as described before, food was restricted. In the paw-reaching-for-food test, rats were required to extend a forelimb through the hole, grasp and retrieve a pellet from the groove, take it to the mouth, and eat it, as Figure 12 shows.

Each time an animal succeeded in eating a pellet without dropping it was counted as a successful response. Dropping the pellet after grasping or raking it was considered as an unsuccessful response.

Each experimental animal was placed in the test cage in individual daily sessions lasting 3 min for 10–12 sessions (in the presurgical phase), for quantifying the number of successful and unsuccessful responses with both paws, and the preferred paw (right or left) of each animal was established. The total number of responses (successful and unsuccessful with both paws), and the percentage of successful responses with the preferred paw with regard to the total number of responses were scored. Furthermore, the paw-reaching test was used after inducing the injury (for testing the efficiency of it) and during the rehabilitative therapy as well (Figure 11A).

### 4.2. Frontal Motor Cortex Ablation

**Figure 13:**
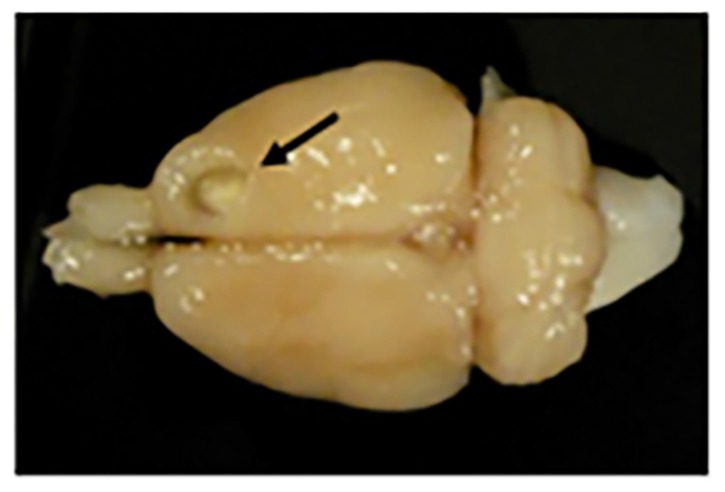
Motor frontal cortex ablation. Photograph of a rat brain with a motor cortex ablation (shown with a black arrow). At the bottom of the lesion is a slightly paler shade that corresponds to the corpus callosum (or white matter).

Under deep anesthesia with Equithesin, each rat was positioned in a stereotaxic apparatus and an incision was made over the forehead to expose the skull. Animals were randomly divided into two groups. One group (*n* = 15) was subjected to a unilateral frontal motor cortex lesion. The other group (*n* = 5) was sham-operated. Animals were lesioned by aspiration in the motor cortex contralateral to the preferred paw or sham-operated. Cortical ablation was performed at the coordinates indicated in previous studies [35,36], to remove the forelimb area of the motor cortex. A craniotomy was made unilaterally from 1 to 4 mm anterior to Bregma, and 1 to 3.5 mm lateral to the midline. Corpus callosum established the ventral limits of the lesion. Using an operating microscope, meninges were removed, and a glass pipette, connected to an aspiration pump, was introduced into the cortex to remove the tissue. The typical size and localization of cortical ablation resulting from this method has been described previously [35,36], and is depicted in Figure 13.

Control animals underwent the same surgical procedure in the motor cortex contralateral to the preferred paw, but no cortical ablation was induced in them (sham-operation). The effectiveness of the lesion was verified at 7 dpi; the paw-reaching test established whether the lesion had been effective: animals began to use their non-preferred paw for reaching food or the percentage of successful responses with the preferred paw was significantly decreased regarding previous values in the presurgical phase.

### 4.3. Treatment with GH and Rehabilitative Therapy

The injured animals were treated with GH (LGH1 group) or vehicle (LV1 group) immediately after motor cortex ablation. GH (rhGH, Saizen, Merck, Madrid, Spain; 0.15 mg/kg/day, s.c) was administered during five consecutive days commencing at 7 h post-ablation (LGH1) (Figure 11A). The other group of injured animals (LV1) received vehicle (0.1 M phosphate-buffered saline, pH 7.4, PBS), following the same temporal pattern as the animals treated with GH (Figure 11A). For control purposes, the group of sham-operated animals received vehicle (CV1 group).

Rehabilitative therapy with the forced use of the affected paw, was performed as described in our previous studies [35,36,41]; it was carried out during nine consecutive days in daily sessions of 3 min, and it was applied to all animals (including sham-operated controls) (Figure 11A). Rehabilitative therapy began at 8 dpi, and the animals were sacrificed at 25 dpi (Figure 11A).

### 4.4. BrdU Administration

To analyze cell proliferation, 5-Bromo-2’-deoxyuridine (BrdU) was used. BrdU is a cell cycle marker used in the adult rodent brain [57]; it is a thymidine analog that is incorporated into the newly synthesized DNA. In animals that were sacrificed at 25 dpi, to evaluate the functional effect of the GH treatment (Figure 11A), BrdU was administered together with 5-fluoro-2’-deoxyuridine (FdU), an inhibitor of thymidine synthesis, to overcome the toxic and mutagenic effects of BrdU. The dividing cells show greater avidity for BrdU when the availability of thymidine is limited; therefore, lower doses of BrdU can be used if combined with FdU [58], overcoming the mutagenic effects of BrdU and poor incorporation into the dividing DNA, therefore allowing the labeling of all S-phase cells [59,60]. No toxic or mutagenic effects of BrdU have been detected in animals injected with the BrdU/FdU mixture [61,62].

BrdU was dissolved in water at a concentration of 16 mg/mL. Animals received a daily injection of BrdU/FdU (30 mg/kg BrdU/3 mg/kg FdU, Merck, Madrid, Spain; ip) for four days. Injections commenced on day 1 after the lesion. No toxic effects of BrdU were detected along the 25 days of the experimental protocol.

In previous studies, we demonstrated that the lesion of hippocampus with kainic acid stimulated the proliferation of neural precursors in the hippocampus, and that this effect was significantly enhanced by the administration of GH [24]. In view of these findings, in this study some injured rats treated with vehicle (LV2) or GH (LGH2) were injected with BrdU using a saturation protocol (100 mg/kg), as in our previous study [24], and euthanized at 5 dpi (Figure 11B); these animals did not receive rehabilitative therapy before being sacrificed.

### 4.5. Immunofluorescence

To analyze the effect of GH on cortical cell proliferation after injury, at 5 dpi, some rats (*n* = 5, Figure 11B) were euthanized by intraperitoneal injection of Equithesin, and perfused transcardially with saline (0.9% NaCl) followed by a fixing solution (4% paraformaldehyde in 0.1 M sodium phosphate buffer, pH 7.4, PBS). Brains were dissected, post-fixed in the same fixative and cryoprotected in 30% sucrose in PBS. Brains were cut in a freezing microtome, and 40-μm-thick coronal sections were collected sequentially in 24-well plates. For BrdU staining, sections were washed with PBS three times, denatured (2 N HCl) for 30 min at 37 °C, neutralized with several rinses with 0.1 M borate buffer, pH 8.5 for 30 min, and washed with PBS three more times. Following several rinses with PBS, sections were incubated in 0.1% sodium borohydride (NaBH_4_) in PBS, to inactivate the autofluorescence, overnight at 4 °C. After several washes, the sections were incubated in a primary antibody solution: mouse anti-BrdU 1:100 (Abcam, ab136650, Cambridge, UK) and rabbit anti-doublecortin 1:1000 (Abcam, ab18723) in PBS with 10% goat normal serum and 0.3% Triton X-100, overnight, at 4 °C. The specificity of the labeling was verified in the control sections incubated without the primary antibodies, in them no specific labeling was observed. The sections were washed three times in PBS, and incubated with corresponding Cy2 and Cy3 conjugated, IgG, secondary antibodies, at 1:500 (Jackson Laboratories, Bar Harbor, ME, USA), in PBS with 10% goat normal serum and 0,3% Triton X-100, for 2 h at room temperature, in darkness. The sections were rinsed with PBS, mounted on slides and covered with Fluoroshield antifade reagent with DAPI (4,6-diamidino-2-phenylindole; F6057, Merck, Madrid, Spain).

### 4.6. Immunohistochemistry

On completion of the rehabilitative therapy, at 25 dpi, rats were euthanized by i.p. injection of Equithesin and perfused transcardially, as indicated before (Figure 11A). Brains were dissected, post-fixed in the same fixative, and cryoprotected in 30% sucrose in PBS. Brains were cut in a freezing microtome, and 40-μm-thick coronal sections were taken throughout the brain. Free-floating brain sections were used for immunohistochemical studies.

Sections were first incubated in 2N HCl and underwent several washes in borate buffer, as indicated previously for BrdU staining. Following several rinses with Tris-phosphate buffer saline, pH 7.4 (TPBS), sections were incubated in a block solution. The block solution included 3% horse normal serum and 0.2% Triton X-100 in TPBS. Then, the sections were incubated in primary antibody solution (mouse anti-BrdU, Abcam, 1:100 in TPBS with 1.5% horse normal serum) for 48 h, at 4 °C. After incubation with the primary antibody, the sections were rinsed several times in TPBS and then incubated in 0.3% hydrogen peroxide in TPBS for 12 min to inactivate the activity of the endogenous peroxidase. After several rinses with TPBS, sections were placed in the secondary antibody (1:200 biotinylated anti-mouse, IgG; Vector Laboratories, Burlingame, CA, USA) in TPBS. Sections were rinsed several times in TPBS and incubated at room temperature for 2 h in a horseradish peroxidase complex (ABC kit, Vector Laboratories). Immunoreactivity was then visualized using 3-3 ‘diaminobenzidine tetrahydrochloride (DAB). Each run of immunohistochemical processing included tissue from all groups to decrease the contribution of the effects of batches to immunostaining variability. The specificity of the labeling was verified in the control sections incubated without the primary antibody, in which no specific labeling was observed. Sections were mounted onto slides and cover-slipped with Entellan.

For nestin and actin detection, coronal sections were incubated in 0.3% hydrogen peroxide in TPBS for 12 min, at room temperature, to inactivate the activity of endogenous peroxidase. They were then subjected to several washes of TPBS, and then incubated in a block solution to avoid binding to non-specific proteins. The block solution was composed of 0.2% Triton X-100 and 3% horse normal serum in TPBS. Sections were incubated in a primary antibody solution (TPBS with 1.5% horse normal serum). The primary antibodies were anti-nestin (mouse, clone Rat-401, 1:90; Chemicon, Feltham, UK) or anti-actin (polyclonal rabbit antibody, 0.2 µg/mL; Merck). Incubations with the primary antibody were performed overnight, at room temperature (nestin) or at 4 °C (actin). After incubation with the primary antibody, the sections were rinsed several times in TPBS and then placed in the secondary antibody (1:200 biotinylated anti-mouse or anti-rabbit, IgG; Vector Laboratories) in TPBS. Sections were rinsed several times in TPBS and incubated at room temperature for 2 h in a horseradish avidin‒peroxidase complex (ABC kit, Vector Laboratories). Immunoreactivity was then visualized using DAB. The specificity of antibody binding was verified with tissue sections incubated without primary antibody, in which no specific labeling was observed. The nestin and actin expression were restricted to the striatum and thalamus, subcortical brain areas involved in the motor activity.

### 4.7. Quantitative Analysis

Four animals (1 LGH2, 1 LV2, 1 LGH1, and 1 LV1) were analyzed at each time point after the injury. Five to 11 40-µm-thick coronal sections per brain were selected across the region of interest in each animal (from Bregma +4 mm to Bregma +1 mm for perilesional cortical BrdU studies). A systematic stereologic approach was used to count BrdU+ cells, in the perilesional (M1) motor cortex with the 10x objective. Cell counts were performed using ImageJ software (National Institutes of Health, Bethesda, MD, USA) and compiled and analyzed. Counting was performed by a blinded observer. Nestin reaction intensity was determined as arbitrary units (AU) of gray level (scale between 0, representing absolute white, and 255, indicating absolute black) with ImageJ free viewer software (National Institutes of Health).

### 4.8. Statistical Analysis

The data analysis was performed using the Statview and SPSS programs. The group of animals treated with GH was compared to the group treated with vehicle and the control sham-operated group. We compared the total number of responses (successful + unsuccessful with both paws) and the percentage of successful responses with the preferred paw with respect to the total number of responses. Fine motor skills results were analyzed by two-way (group and session) analysis of variance (ANOVA). When global ANOVA showed a significant difference among groups (*p* ≤ 0.05), partial ANOVA comparing the different groups in each session was performed. The Bonferroni post hoc test (*p* ≤ 0.0167) was used to compare the individual means. Student’s *t*-test was used to compare the means in immunofluorescence and immunohistochemistry studies.

## 5. Conclusions

From our data, we can conclude that GH is a very important factor in the repair of an injured brain, but its administration has to be accompanied by rehabilitative therapy in order to achieve functionally significant improvements. GH administration and rehabilitation induce significant nestin re-expression in the undamaged contralateral motor cortex, but this effect seems to depend on mechanisms of neural plasticity in resident cortical cells, and not on neurogenesis. GH administration also increases the expression of nestin in the striatum of injured rats, which may be involved in the functional recovery after frontal motor cortex ablation. This damage, by itself, induces the expression of nestin and actin in the activated microglia/macrophage cells in the VA/VL thalamic complex ipsilateral to the lesion produced, which probably plays a key role in the remodeling of the cellular cytoskeleton and allows the development of the compensatory brain plasticity responsible for the observed functional improvements. We cannot discount the possibility that some of the changes observed could be due to the induction by GH of the expression of some neurotrophic factors, such as IGF-I, GDNF or BDNF, since these factors play an important role in brain repair after an injury [17,63].

## References

[1] Marklund N., Fulp C.T., Shimizu S., Puri R., McMillan A., Strittmatter S.M., McIntosh T.K. (2006). Selective temporal and regional alterations of Nogo-A and small proline-rich repeat protein 1A (SPRR1A) but not Nogo-A receptor (NgR) occur following traumatic brain injury in the rat. Exp. Neurol..

[2] Nudo R.J. (2006). Mechanisms for recovery of motor function following cortical damage. Curr. Opin. Neurobiol..

[3] Nudo R.J. (2003). Adaptive plasticity in motor cortex: Implications for rehabilitation after brain injury. J. Rehab. Med..

[4] Yang G., Pan F., Gan W.B. (2009). Stably maintained dendritic spines are associated with lifelong memories. Nature.

[5] Conner J.M., Chiba A.A., Tuszynski M.H. (2005). The basal forebrain cholinergic system is essential for cortical plasticity and functional recovery following brain injury. Neuron.

[6] Dancause N., Barbay S., Frost S.B., Plautz E.J., Chen D., Zoubina E.B., Stowe A.M., Nudo R.J. (2005). Extensive cortical rewiring after brain injury. J. Neurosci..

[7] Li S., Overman J.J., Katsman D., Kozlov S.V., Donnelly C.J., Twiss J.L., Giger R.J., Coppola G., Geschwind D.H., Carmichael S.T. (2010). An age-related sprouting transcriptome provides molecular control of axonal sprouting after stroke. Nat. Neurosci..

[8] Ohab J.J., Carmichael S.T. (2008). Poststroke neurogenesis: Emerging principles of migration and localization of immature neurons. Neuroscientist.

[9] Chu C.J., Jones T.A. (2000). Experience-dependent structural plasticity in cortex heterotopic to focal sensorimotor cortical damage. Exp. Neurol..

[10] Jones T.A., Schallert T. (1994). Use-dependent growth of pyramidal neurons after neocortical damage. J. Neurosci..

[11] Jones T.A., Kleim J.A., Greenough W.T. (1996). Synaptogenesis and dendritic growth in the cortex opposite unilateral sensorimotor cortex damage in adult rats: A quantitative electron microscopic examination. Brain Res..

[12] Jones T.A., Schallert T. (1992). Overgrowth and pruning of dendrites in adult rats recovering from neocortical damage. Brain Res..

[13] Jones T.A., Adkins D.L., Cramer S.C.N.R. (2010). Behavioral influences on neuronal events after stroke. Brain Repair After Stroke.

[14] Brown C.E., Wong C., Murphy T.H. (2008). Rapid morphologic plasticity of peri-infarct dendritic spines after focal ischemic stroke. Stroke.

[15] Brown C.E., Boyd J.D., Murphy T.H. (2010). Longitudinal in vivo imaging reveals balanced and branch-specific remodeling of mature cortical pyramidal dendritic arbors after stroke. J. Cereb. Blood Flow Metab..

[16] Lobie P.E., Zhu T., Graichen R., Goh E. (2000). Growth hormone, insulin-like growth factor I and the CNS: Localization, function and mechanism of action. Growth Horm. IGF Res..

[17] Devesa J., Almengló C., Devesa P. (2016). Multiple Effects of Growth Hormone in the Body: Is it Really the Hormone for Growth?. Clin. Med. Insights Endocrinol. Diabetes.

[18] Scheepens A., Sirimanne E.S., Breier B.H., Clark R.G., Gluckman P.D., Williams C.E. (2001). Growth hormone as a neuronal rescue factor during recovery from CNS injury. Neuroscience.

[19] Shin D.H., Lee E., Kim J.W., Kwon B.S., Jung M.K., Jee Y.H., Kim J., Bae S.R., Chang Y.P. (2004). Protective effect of growth hormone on neuronal apoptosis after hypoxia-ischemia in the neonatal rat brain. Neurosci. Lett..

[20] Aberg N.D., Brywe K.G., Isgaard J. (2006). Aspects of growth hormone and insulin-like growth factor-I related to neuroprotection, regeneration, and functional plasticity in the adult brain. Sci. World J..

[21] Isgaard J., Aberg D., Nilsson M. (2007). Protective and regenerative effects of the GH/IGF-I axis on the brain. Minerva Endocrinol..

[22] Christophidis L.J., Gorba T., Gustavsson M., Williams C.E., Werther G.A., Russo V.C., Scheepens A. (2009). Growth hormone receptor immunoreactivity is increased in the subventricular zone of juvenile rat brain after focal ischemia: A potential role for growth hormone in injury-induced neurogenesis. Growth Horm. IGF Res..

[23] Pathipati P., Surus A., Williams C.E., Scheepens A. (2009). Delayed and chronic treatment with growth hormone after endothelin-induced stroke in the adult rat. Behav. Brain Res..

[24] Devesa P., Reimunde P., Gallego R., Devesa J., Arce V.M. (2011). Growth hormone (GH) treatment may cooperate with locally-produced GH in increasing the proliferative response of hipocampal progenitors to kainate-induced injury. Brain Inj..

[25] Li R.C., Guo S.Z., Raccurt M., Moudilou E., Morel G., Brittian K.R., Gozal D. (2011). Exogenous growth hormone attenuates cognitive deficits induced by intermittent hypoxia in rats. Neuroscience.

[26] Alba-Betancourt C., Luna-Acosta J.L., Ramírez-Martínez C.E., Avila-González D., Granados-Ávalos E., Carranza M., Martínez-Coria H., Arámburo C., Luna M. (2013). Neuro-protective effects of growth hormone (GH) after hypoxia-ischemia injury in embryonic chicken cerebellum. Gen. Comp. Endocrinol..

[27] High W.M., Briones-Galang M., Clark J.A., Gilkison C., Mossberg K.A., Zgaljardic D.J., Masel B.E., Urban R.J. (2010). Effect of growth hormone replacement therapy on cognition after traumatic brain injury. J. Neurotrauma.

[28] Reimunde P., Quintana A., Castañón B., Casteleiro N., Vilarnovo Z., Otero A., Devesa A., Otero-Cepeda X.L., Devesa J. (2011). Effects of growth hormone (GH) replacement and cognitive rehabilitation in patients with cognitive disorders after traumatic brain injury. Brain Inj..

[29] Moreau O.K., Cortet-Rudelli C., Yollin E., Merlen E., Daveluy W., Roseaux M. (2013). Growth hormone replacement therapy in patients with traumatic brain injury. J. Neurotrauma.

[30] Devesa J., Devesa P., Reimunde P., Arce V., Agrawal A. (2012). Growth hormone and kynesitherapy for brain injury recovery. Brain Injury—Pathogenesis, Monitoring, Recovery and Management.

[31] Devesa J., Reimunde P., Devesa P., Barberá M., Arce V. (2013). Growth hormone (GH) and brain trauma. Horm. Behav..

[32] Arce V.M., Devesa P., Devesa J. (2013). Role of growth hormone (GH) in the treatment of neural diseases: From neuroprotection to neural repair. Neurosci. Res..

[33] Devesa J., Díaz-Getino G., Rey P., García-Cancela J., Loures I., Nogueiras S., de Mendoza A.H., Salgado L., González M., Pablos T. (2015). Brain recovery after a plane crash: Treatment with growth hormone (GH) and neurorehabilitation: A case report. Int. J. Mol. Sci..

[34] Dubiel R., Callender L., Dunklin C., Harper C., Bennett M., Kreber L., Auchus R., Diaz-Arrastia R. (2018). Phase 2 Randomized, Placebo-Controlled Clnical Trial of Recombinant Human Growth Hormone (rhGH) During Rehabilitatio From Traumatic Brain Injury. Front. Endocrinol..

[35] Heredia M., Fuente A., Criado J., Yajeya J., Devesa J., Riolobos A.S. (2013). Early growth hormone treatment promotes relevant motor functional improvement after severe frontal cortex lesion in adult rats. Behav. Brain Res..

[36] Heredia M., Palomero J., de la Fuente A., Criado J.M., Yajeya J., Devesa J., Devesa P., Vicente-Villardón J.L., Riolobos A.S. (2018). Motor Improvement of Skilled Forelimb Use Induced by Treatment with Growth Hormone and Rehabilitation is Dependent on the Onset of the Treatment after Cortical Ablation. Neural. Plast..

[37] Lendahl U., Zimmerman L.B., McKay R.D.G. (1990). CNS stem cells express a new class of intermediate filament protein. Cell.

[38] Dahlstrand J., Lardelli M., Lendahl U. (1995). Nestin mRNA expression correlates with the central nervous system progenitor cell state in many, but not all, regions of developing central nervous system. Dev. Brain Res..

[39] McKay R. (1997). Stem cells in the central nervous system. Science.

[40] Rao M.S. (1999). Multipotent and restricted precursors in the central nervous system. Anatom Rec.

[41] Riolobos A.S., Heredia M., de la Fuente J.A., Criado J.M., Yajeya J., Campos J., Santacana M. (2001). Functional recovery of skilled forelimb use in rats obliged to use the impaired limb after grafting of the frontal cortex lesion with homotopic fetal cortex. Neurobiol. Learn Mem..

[42] Walker T.L., Yasuda T., Adams D.J., Bartlett P.F. (2007). The doublecortin-expressing population in the developing and adult brain contains multipotential precursors in addition to neuronal-lineage cells. J. Neurosci..

[43] Parent J.M. (2003). Injury-induced neurogenesis in the adult mammalian brain. Neuroscientist.

[44] Susarla B.T., Villapol S., Yi J.H., Geller H.M., Symes A.J. (2014). Temporal patterns of cortical proliferation of glial cell populations after traumatic brain injury in mice. ASN Neuro.

[45] Wilson C.J. (1987). Morphology and synaptic connections of crossed corticostriatal neurons in the rat. J. Comp. Neurol..

[46] Kartje G.L., Schultz M.K., Lopez-Yunez A., Schnell L., Schwab M.E. (1999). Corticostriatal plasticity is restricted by myelin-associated neurite growth inhibitors in the adult rat. Ann. Neurol..

[47] McNeill T.H., Mori N., Cheng H.W. (1999). Differential regulation of the growth-associated proteins, GAP-43 and SCG-10, in response to unilateral cortical ablation in adult rats. Neuroscience.

[48] Shen C.C., Yang Y.C., Chiao M.T., Cheng W.Y., Tsuei Y.S., Ko J.L. (2010). Characterization of endogenous neural progenitor cells after experimental ischemic stroke. Curr. Neurovasc. Res..

[49] Szele F.G., Alexander C., Chesselet M.F. (1995). Expression of molecules associated with neuronal plasticity in the striatum after aspiration and thermocoagulatory lesions of the cerebral cortex in adult rats. J. Neurosci..

[50] Groenewegen H.J., Witter M.P., Paxinos G. (2004). Thalamus. The Rat Nervous System.

[51] Jones E.G. (2007). The Thalamus.

[52] Lam Y.-W., Sherman S.M. (2015). Functional organization of the motor reticulothalamic pathway. J. Neurophysiol..

[53] Biane J.S., Takashima Y., Scanziani M., Conner J.M., Tuszynski M.H. (2016). Thalamocortical projections onto behaviorally relevant neurons exhibit plasticity during adult motor learning. Neuron.

[54] Acarin L., González B., Hidalgo J., Castro A.J., Castellano B. (1999). Primary cortical glial reaction versus secondary thalamic glial response in the excitotoxically injured young brain: Astroglial response and metallothionein expression. Neuroscience.

[55] Loane D.J., Byrnes K.R. (2010). Role of microglia in neurotrauma. Neurotherapeutics.

[56] Plantier M., Der Terrossian E., Represa A. (1998). Beta-actin immunoreactivity in rat microglial cells: Developmental pattern and participation in microglial reaction after kainate injury. Neurosci. Lett..

[57] Valero J., Weruaga E., Murias A.R., Recio J.S., Alonso J.R. (2005). Proliferation markers in the adult rodent brain: Bromodeoxyuridine and proliferating cell nuclear antigen. Brain Res Brain Res Protoc.

[58] Ellwart J., Dörmer P. (1985). Effect of 5-fluoro-2’-deoxyuridine (FdUrd) on 5-bromo-2’-deoxyuridine (BrdUrd incorporation into DNA measured with a monoclonal BrdUrd antibody and by the BrdUrd/Hoechst quenching effect. Cytometry.

[59] Matthews D.A., Villafranca J.E., Janson C.A., Smith W.W., Welsh K., Freer S. (1990). Stereochemical mechanism of action for thymidilate synthase based on the X-ray structure of the covalent inhibitory ternary complex with 5-fluoro-2’-deoxyuridylate and 5,10-methylene-tetrahydrofolate. J. Mol. Biol..

[60] Mancini W.R., Stetson P.L., Lawrence T.S., Wagner J.G., Greenberg H.S., Ensminger W.D. (1991). Variability of 5-bromo-2’-deoxyuridine incorporation into DNA of human glioma cell lines and modulation with fluoropyrimidines. Cancer.

[61] Kolb B., Pedersen B., Ballermann M., Gibb R., Whishaw I.Q. (1999). Embryonic and postnatal injections of bromodeoxyuridine produce age-dependent morphological and behavioral abnormalities. J. Neurosci..

[62] Morris S.M. (1991). The genetic toxicology of 5-bromodeoxyuridine in mammalian cells. Mutat. Res..

[63] Lux X., Hagg T. (1997). Glial cell line-derived neurotrophic factor prevents death, but not reductions in tyrosine hydroxylase, of injured nigrostriatal neurons in adult rats. J Comp Neurol.

